# Current Status of Molecularly Targeted Therapeutics in Blood Cancers

**DOI:** 10.3390/ijms262110512

**Published:** 2025-10-29

**Authors:** Caitlin Kumala, Lynh Vu, Tamer E. Fandy

**Affiliations:** Paul L. Foster School of Medicine, Texas Tech University Health Sciences Center El Paso, El Paso, TX 79905, USA; ckumala@ttuhsc.edu (C.K.); lynvu@ttuhsc.edu (L.V.)

**Keywords:** blood cancer, tyrosine kinase inhibitors (TKIs), immune therapies, PROTACs, FLT3 inhibitors, proteasome inhibitors

## Abstract

Blood cancer is characterized by the uncontrolled growth of blood cells in the bone marrow or in the lymphatic system. Chemotherapy is still considered the first line of treatment in several types of blood cancer despite its adverse effects. Recent advances in understanding the pathology and genomic changes in these cancers have led to the discovery of novel drug targets and the development of new therapeutic agents. In this review, we will discuss the mechanisms of action and clinical utility of several classes of targeted therapy used in blood cancers, including inhibitors of different types of tyrosine kinase enzymes (BCR-ABL, FLT3 and BTK), BCL-2 inhibitors, phosphoinositide 3-kinase inhibitors, nuclear export inhibitors, immune therapies (monoclonal antibodies, radioimmunoconjugates, chimeric antigen receptor T-cells, immune checkpoint inhibitors, and bispecific antibodies), and proteasome-dependent drugs (proteasome inhibitors and proteolysis targeting chimeras). Further advances in identifying distinct molecular subgroups in blood cancers will offer more opportunities for novel targeted therapies and more personalized medicine approaches.

## 1. Introduction

The inadequacy of treatment options for the older or frail patients with hematologic malignancies is still an obstacle in the treatment of these patient populations. The current advances in understanding the molecular changes in hematologic malignancies accelerated the discovery and development of novel therapeutic agents such as tyrosine kinase inhibitors, antibodies targeting cell surface markers overexpressed on tumor cells, inhibitors of checkpoint proteins that inhibit the immune system from attacking tumor cells, genetically engineered T-cells that recognize and kill tumor cells, inhibitors of the overexpressed anti-apoptotic BCL-2 in several myeloid and lymphoid tumors, and targeted degradation of oncogenes using proteolysis targeting chimeras (PROTACs). Epigenetic therapy has evolved over the last 20 years, targeting DNA methyltransferases, histone deacetylases, the menin-KMT2A complex and isocitrate dehydrogenases (IDH1/2). Epigenetic therapy will not be discussed here because of space limitations, and the reader is directed to other comprehensive and insightful sources instead [1].

## 2. Tyrosine Kinase Inhibitors

The introduction of tyrosine kinase inhibitors (TKIs) in the late 1980s marked a paradigm shift in the treatment of hematologic malignancies, particularly for acute lymphoblastic leukemia (ALL) and chronic myeloid leukemia (CML). TKIs, such as imatinib, inhibited the constitutively active breakpoint cluster region-Abelson (BCR-ABL) tyrosine kinase by competing with adenosine triphosphate (ATP) for its binding site [2]. The next generations of TKIs were developed to counteract resistance mechanisms. Table 1 compares the potency of different generations of TKIs in inhibiting BCR-ABL and shows the increased potency of the second and third generations compared to imatinib. Mutations in the ABL1 kinase domain were linked to increased TKI resistance. Furthermore, recent studies have shown that intrinsic mechanisms also played a role in TKI resistance. For instance, imatinib resistance was linked to deletions in key regulatory genes, including IKZF1, CDKN2A/B, PAX5, and IKZF1^plus^ (co-occurrence of IKZF1 deletions with deletions in PAX5, CDKN2A/2B, or PAR1). Additionally, mutations in ZEB2, SETD2, SH2B3, and CRLF2 were associated with reduced imatinib sensitivity [3].

### 2.1. First-Generation TKI: Imatinib

Imatinib was the first clinically approved TKI that selectively inhibited BCR-ABL, stem cell factor receptor c-KIT, and platelet-derived growth factor receptor (PDGFR) [2]. Originally indicated for CML treatment, imatinib was also approved for gastrointestinal stromal tumors (GISTs) with c-KIT mutation [3]. Imatinib remained the current standard of care due to its high efficacy rate (around 90% five-year survival rates) and low toxicity profile. Common adverse effects were typically mild to moderate, ranging from edema, cytopenia, and hepatotoxicity [4].

Despite the positive clinical outcome associated with the use of imatinib in the treatment of Philadelphia chromosome-positive chronic myeloid leukemia (Ph+ CML) and Philadelphia chromosome-positive acute lymphoblastic leukemia (Ph+ ALL), relapse due to drug resistance was a major concern. Kinase domain point mutations, like the T315I of the fusion protein BCR-ABL, were identified as the main resistance mechanism, which stimulated the development of next-generation TKIs to overcome acquired resistance [5]. On the other hand, intrinsic mechanisms of resistance to TKIs were also observed [3].

**Table 1:** **Comparison of the potency of different TKI generations in inhibiting BCR-ABL.** IC_50_ indicates the half-maximal inhibitory concentration expressed in nanomolar (nM).

Drug Name	Generation	Potency (IC_50_) in nM	References
Imatinib	First	100	[6]
Dasatinib	Second	1	[6]
Nilotinib	Second	20	[6]
Bosutinib	Second	1	[7]
Radotinib	Second	34	[8]
Ponatinib	Third	0.37	[6]
Olverembatinib	Third	0.5	[9]
Asciminib	Third	3.8	[10]
Vodobatinib	Third	7	[11]

The resistant patients showed effective inhibition and reduced dependence on BCR-ABL signaling, which implied an alternative resistance pathway. Consistently, the co-deletions of genes related to B-cell differentiation and cell cycle like *IKZF1*, *PAX5* and *CDKN2A/B* were associated with ex vivo imatinib resistance. Moreover, the intrinsic ex vivo resistance to imatinib demonstrated cross-resistance toward the second- and third-generation TKIs. The above evidences supported a resistance mechanism different from BCR-ABL mutations and suggested that the use of alternative TKIs for patients with intrinsic imatinib resistance was not an effective approach.

### 2.2. Second-Generation TKIs: Dasatinib, Nilotinib, Bosutinib, and Radotinib

**Figure 1:**
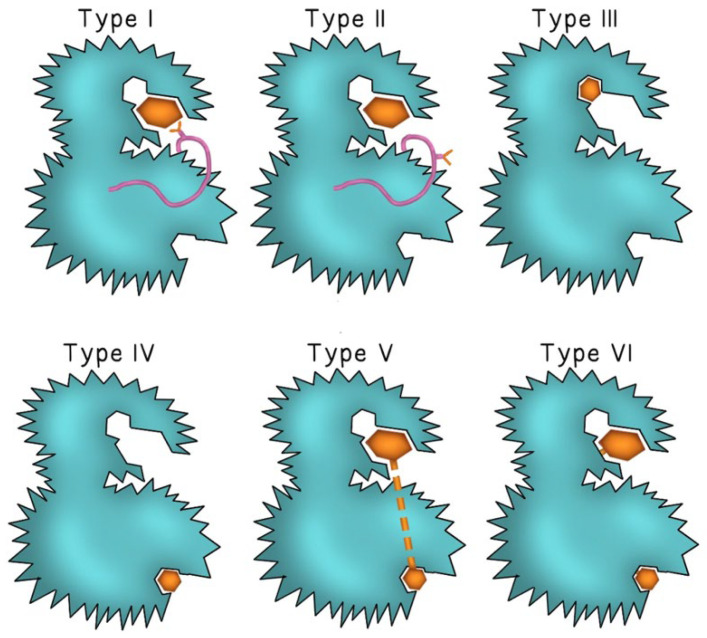
**The binding mode of different TKI generations.** Type I TKIs compete for the ATP binding site of the kinase enzyme and binds to the “DFG-in” active conformation of the enzyme (the pink ribbon represents the Asp-Phe-Gly motif and the Y-shape represents the aspartate amino acid residue of the DFG motif). In Type II TKIs, the inhibitor binds to the ATP binding site of the enzyme in the “DFG-out” inactive conformation (aspartate residue faces outward of the binding site of the enzyme). Type III and type IV TKIs bind to different allosteric sites on the enzyme. Type V and type VI inhibitors are bivalent molecules that bind to both the ATP binding pocket and the distant allosteric site with type VI inhibitors forming irreversible covalent bonds at both sites. The inhibitor molecule is represented by either a small or large brown hexagon.

Second-generation TKIs were designed to counteract TKIs-acquired resistance through mutations in the binding region of BCR-ABL observed with imatinib. Examples include dasatinib and nilotinib, both of which demonstrated efficacy in patients with imatinib-resistant CML [12]. Second-generation TKIs demonstrated increased binding affinities to the ATP binding site of BCR-ABL, allowing for a stronger and more sustained molecular response [12]. Dasatinib also inhibited the Src kinase [13] and was unique in that it could bind to BCR-ABL in both its active (open) and inactive (closed) conformations. In contrast, imatinib and nilotinib bound to a region of the tyrosine kinase enzyme that locked the enzyme in a closed state, preventing the kinase domain from binding to ATP [12]. Figure 1 describes the mode of binding to different sites of the kinase enzyme by different TKI generations. Dasatinib was approved for patients with newly diagnosed imatinib-resistant or intolerant CML, blast-phase CML, as well as Ph+ ALL [14]. Dasatinib’s efficacy in treating solid tumors through inhibition of the Src kinase family remains under investigation [13]. While second-generation TKIs retained activity against most point mutations that conferred resistance to imatinib, they remained inactive against the T315I gatekeeper mutation [15].

Bosutinib functions as a dual Src and Abl kinase inhibitor and is used to treat adults with resistant chronic, accelerated, or blast-phase CML. Bosutinib is effective against most BCR-ABL mutations that conferred resistance to imatinib except for V299L and T315I. Adverse effects were minimal, with mild diarrhea being among the most reported. Additionally, bosutinib demonstrated reduced hematological toxicity compared to other TKIs due to its minimal inhibition of PDGFR and c-KIT [16]. Radotinib is another BCR-ABL inhibitor indicated for chronic-phase CML. It was approved by the Korea Food and Drug Administration but did not receive approval in the US [17]. It is effective against the V299L, F317L, and M351T point mutations [18].

### 2.3. Third-Generation TKIs: Ponatinib, Olverembatinib, Asciminib, and Vamotinib

Third-generation TKIs were developed to address resistance mutations that remained problematic with earlier drugs, most notably the T315I gatekeeper mutation, which was found in 15–20% of patients with CML [12]. Ponatinib is FDA-approved for CML patients with T315I mutation who were resistant or intolerant to at least two prior TKIs. Response-based dosing strategies helped to mitigate cardiovascular toxicities and improved patients’ tolerance to treatment [19]. Clinical trials, such as PACE and OPTIC, have demonstrated ponatinib’s ability to induce strong responses in patients who were resistant to second-generation TKIs. In these studies, response rates and survival outcomes were significantly increased, especially when dose adjustments were performed based on response milestones. However, there was an increased risk of adverse vascular events like arterial occlusion and hypertension [20]. The continued development of third-generation TKIs and rational combination therapies is expected to drive further advancements in the management of CML and Ph+ ALL.

Olverembatinib is a novel third-generation TKI approved in China, in 2021, for the treatment of adult patients with TKI-resistant chronic-phase or accelerated-phase CML with the T315I mutation. Recent clinical trials highlighted its efficacy in generating significant hematologic and cytogenetic responses in patients who were resistant to at least two prior TKIs, with mild side effects such as skin hyperpigmentation and rare grade 3 or 4 adverse events like hypertriglyceridemia and thrombocytopenia [21]. Olverembatinib’s ability to target both phosphorylated and non-phosphorylated forms of BCR-ABL, along with other kinases like KIT and fms-like tyrosine kinase 3 (FLT3), demonstrated its broad therapeutic potential.

Asciminib was the first TKI to target the myristoyl pocket of BCR-ABL [22]. This advantage made treatment with asciminib favorable for patients who were heavily pretreated and developed resistance or intolerance to two or more TKI therapies. Fewer cardiovascular adverse effects were reported compared to other TKIs, along with thrombocytopenia and potential cumulative toxicity in the gastrointestinal tract. Potential resistance mechanisms against asciminib involve mutations at the myristoyl binding site of BCR-ABL and upregulation of the ABCG2 efflux pump with a consequent decrease in asciminib intracellular concentration. Asciminib developed unfavorable drug–drug interactions through the inhibition of drug transporters such as P-gp, BCRP, and OATP1B. For instance, OATP1B is a substrate for statin drugs used to lower blood cholesterol and is essential for the intracellular uptake of statins by liver cells. Inhibition of OATP1B by asciminib led to higher plasma levels of statins and increased risk of statins-induced myopathy.

Vamotinib, also known as PF-114, is a third-generation TKI that was chemically modified to prevent the inhibition of the vascular endothelial growth factor receptor (VEGFR) to reduce its cardiovascular toxicity [23]. In a phase I/II clinical study (NCT02885766) that enrolled 51 patients with resistance to second-generation TKIs or carried a T315I mutation, vamotinib treatment led to a complete hematologic response in 14 of 30 participants, a major cytogenetic response in 14 of 44, a complete cytogenetic response in 10 of 50, and a major molecular response in 7 of 51. Overall, vamotinib was concluded to be an acceptable alternative for patients who were resistant or intolerant to more than two TKIs.

## 3. FLT3 Inhibitors

The discovery of activating mutations in the FLT3 receptor in approximately 30% of acute myeloid leukemia (AML) patients identified FLT3 as a therapeutic target [3]. FLT3, located on chromosome 13q12, is composed of five domains—an extracellular domain, juxtamembrane domain, tyrosine kinase domain, kinase insert, and a C-terminal intracellular domain. FLT3 is involved in hematopoietic stem and progenitor cell survival, proliferation, and differentiation through downstream receptor tyrosine kinase signaling pathways [24]. Mutations in FLT3 include internal tandem duplication (ITD) in the juxtamembrane domain and point mutations in the tyrosine kinase domain (TKD) [25]. Based on target specificity, FLT3 inhibitors were categorized as first- or second-generation, whereas their interaction mechanism with FLT3 classified them as type I or type II inhibitors [26]. Type I inhibitors bind to the ATP binding pocket when the receptor FLT3 is in its active DFG-in conformation, allowing inhibition of both FLT3-ITD and FLT3-TKD mutations. Type II inhibitors bind to a hydrophobic pocket next to the ATP site, accessible only in the inactive DFG-out conformation, and thus can only inhibit FLT3-ITD [27]. Table 2 compares the potency of FLT3 inhibitors from different generations and shows gilteritinib as the most potent inhibitor of FLT3.

FLT3-ITD was associated with poor prognosis in AML compared to FLT3-TKD mutations or no FLT3 mutation. Standard therapy had a limited efficacy of a 30–40% cure rate, and it was found that FLT3-ITD mutations had a high relapse rate and short relapse-free duration in AML patients, thus decreasing post-chemotherapy and post-transplant survival rates [28]. In addition, FLT3-ITD was an independent marker associated with high leukemic burden and leukocytosis. The prognosis of FLT3-TKD mutations was less clear than FLT3-ITD mutations, and appeared to be dependent on other factors such as co-occurring mutations and cytogenetic changes [29].

The contribution of FLT3 mutations to leukemogenesis and their role as driver or passenger mutation is controversial. In support of the driver mutation hypothesis, FLT3 mutations often occurred early in clonal evolution and were associated with high leukemic burden. On the other hand, the limited success of FLT3 inhibitors as monotherapy—illustrated by suboptimal outcomes with midostaurin alone and the failure of lestaurtinib when combined with chemotherapy—suggests that FLT3 may not be the sole pathogenic driver in all cases [30]. These findings highlight the need for combination therapies and a deeper understanding of co-occurring mutations and resistance mechanisms in FLT3-mutated AML [24].

### 3.1. First-Generation FLT3 Inhibitors: Lestaurtinib, Tandutinib, Midostaurin, Sorafenib, and Sunitinib

The first-generation FLT3 inhibitors include lestaurtinib, tandutinib, midostaurin, sorafenib, and sunitinib. They were originally developed as multi-kinase inhibitors with activity against FLT3. Unfortunately, they lacked specificity, demonstrated low efficacy, and were associated with significant adverse effects when administered as monotherapy. Lestaurtinib is a staurosporine analog type I inhibitor with broad specificity. It is no longer in development due to poor clinical efficacy and toxicity profile. Tandutinib is a type II inhibitor that inhibits FLT3, PDGFR, and c-KIT. It exerted its antileukemic effects by inducing apoptosis through modulation of key intracellular signaling cascades, particularly the mitogen-activated protein kinase (MAPK) and phosphoinositide 3-kinase (PI3K) pathways [31]. Midostaurin is another staurosporin analog type I inhibitor with multi-kinase domain inhibition. In addition to FLT3 inhibition, midostaurin also demonstrated VEGFR inhibitor activity, which increased its toxicity. It is currently approved for adults with newly diagnosed FLT3 mutation-positive AML in combination with the standard of care of cytarabine and daunorubicin [12].

Sorafenib inhibited multiple receptor tyrosine kinases involved in tumor signaling and apoptosis including FLT3, RET, KIT, VEGFR, and PDGFR by directly blocking auto-phosphorylation [12,32]. Sorafenib inhibited the growth of hepatocellular carcinoma (HCC), renal cell carcinoma (RCC), and ductal thyroid carcinoma human tumor xenografts in immunocompromised mice. Suppression of tumor angiogenesis and increase in tumor apoptosis were observed in models of HCC and RCC upon sorafenib treatment. However, sorafenib is not approved for AML or any type of hematologic malignancies due to lack of efficacy. Sunitinib is another multitargeted TKI of VEGFR, PDGFR, KIT, FLT, and RET [33]. Sunitinib is currently FDA-approved for RCC, gastrointestinal stromal tumor, and pancreatic neuroendocrine tumors [34]. A phase I study with sunitinib showed partial remissions of short duration in AML but also demonstrated high toxicity. The low FLT3 inhibitory activity and short therapeutic response discouraged further development of sunitinib for AML therapy [35].

### 3.2. Second-Generation FLT3 Inhibitors: Quizartinib and Gilteritinib

The development of second-generation FLT3 inhibitors marked a significant advancement in the treatment of FLT3-mutated AML. Second-generation FLT3 inhibitors were designed to selectively target FLT3-ITD mutations. Approximately one-third of AML patients were noted to have ITD mutation, and its presence was linked to poorer outcomes [36]. Second-generation FLT3 inhibitors aimed to overcome resistance mechanisms seen with earlier therapies and improve clinical outcomes.

Quizartinib is a highly active and selective FLT3 inhibitor; however, the responses were suboptimal. In clinical trials, complete remission rates were low and short-lived [36]. Thus, further research was recommended to improve its efficacy through combination therapy. It was FDA-approved in 2023 and indicated for newly diagnosed AML patients with positive FLT3-ITD mutation in combination with standard chemotherapy. Its dose-limiting toxicity is QT prolongation and is only available through a restricted program [37]. Gilteritinib is a type I inhibitor that targets several tyrosine kinase receptors, including FLT3, AXL, and anaplastic lymphoma kinase (ALK). It demonstrated highly selective inhibitory activity against cells with FLT3-ITD and FLT3-TKD mutations, especially FLT3-D835Y and FLT3-ITD-D835Y point mutations, while showing relatively weaker activity against c-KIT. Gilteritinib also demonstrated selective inhibition of AXL, which normally functions to activate FLT3 and contributes to resistance development against FLT3 inhibitors [25].

### 3.3. Novel FLT3 Inhibitors

FLT3 inhibitors are mainly targeted for the treatment of AML with FLT3 mutations. Novel FLT3 inhibitors and multikinase inhibitors are currently in development to further overcome current resistance mechanisms and to limit toxic side effects [38]. Ponatinib is primarily a BCR-ABL inhibitor currently approved for CML and also exhibited type II FLT3-ITD inhibitor activity [39]. Similarly, cabozantinib is approved for medullary thyroid and RCC, but also displayed selective cytotoxic effects to FLT3-ITD mutant cells, including D835 resistance mutations [40]. The use of ponatinib and cabozantinib in AML is still under investigation and none of them received approval for AML treatment. Ibrutinib is primarily approved for chronic lymphocytic leukemia (CLL) but is also shown to inhibit FLT3-ITD in AML cells [41]. Additional emerging FLT3 inhibitors include FF-10101, TTT-3002, G-749, MZH-29, and HM43239 [30]. In particular, FF-10101 is promising as it inhibited both FLT3-ITD and FLT3-TKD, and was among the first inhibitors to form an irreversible covalent bond with FLT3 with consequent sustained inhibition [38].

## 4. Bruton Tyrosine Kinase Inhibitors

Bruton tyrosine kinase (BTK) is a non-receptor tyrosine kinase that is expressed in many cells of hematopoietic lineage, including B-cells, platelets, and myeloid cells [42]. It is integral to the function and maturation of B-cells, as well as modulating the B-cell receptor (BCR) signal transduction pathway [43]. The activation of BCR signaling triggers BTK, which in turn activates downstream pathways such as PI3K/protein kinase B (AKT), phospholipase C, protein kinase C, NF-kB, and the proliferation of malignant B-cells [42]. Accordingly, BTK inhibitors could serve as a rational therapeutic option for treatment of B-cell malignancies.

### 4.1. First-Generation BTK Inhibitor: Ibrutinib

Ibrutinib was originally FDA-approved for patients with relapsed or refractory CLL, including those with the 17p deletion, but has now expanded to be used as first-line treatment for CLL and small lymphocytic lymphoma (SLL). Ibrutinib irreversibly binds covalently to Cys481 residue of BTK and inhibits the phosphorylation of downstream targets with consequent apoptosis of B-cells [44]. Despite its therapeutic efficacy, its lack of BTK selectivity is a drawback associated with undesirable adverse effects. Most notably, off-target inhibition of the C-terminal Src kinase caused atrial fibrillation and hypertension [45,46]. These cardiovascular events may limit or discontinue treatment and highlight the importance of cardiovascular monitoring during ibrutinib therapy [47]. Unlike traditional chemotherapy, ibrutinib did not cause myelosuppression and allowed patients to tolerate longer term therapy [44]. Resistance to ibrutinib developed through mutations within the BTK binding site, or alterations in the BTK pathway [48].

### 4.2. Second-Generation BTK Inhibitors: Acalbrutinib and Zanubrutinib

Second-generation BTK inhibitors such as acalbrutinib and zanubrutinib were designed to be more specific and have minimal off-target interactions [49]. Similarly to ibrutinib, second-generation BTK inhibitors were associated with second primary malignancies including skin cancers and other carcinomas, likely related to off-target EGFR inhibition [50]. While both acalbrutinib and zanubrutinib are FDA-approved for mantle cell lymphoma (MCL), CLL, and SLL, zanubrutnib is also indicated for relapsed or refractory marginal zone lymphoma (MZL) and follicular lymphoma (FL) [51,52].

### 4.3. Third-Generation BTK Inhibitor: Pirtobrutinib

Third-generation BTK inhibitors were designed to counteract drug resistance caused by point mutations like the BTK C481S mutation and to restore effective BTK inhibition [46,53]. Pirtobrutinib is a noncovalent inhibitor of BTK that binds to wild-type BTK and BTK harboring C481 mutations, leading to inhibition of BTK activity. Pirtobrutinib showed dose-dependent antitumor activities in wild-type BTK and BTK C481S mutant mouse xenograft models. Pirtobrutinib was approved in 2023 for relapsed or refractory MCL, CLL, and SLL. Its adverse effects are similar to earlier BTK inhibitors, including risks of cytopenias, hemorrhage, infections, cardiac arrhythmias, hepatotoxicity, and second primary malignancies [54,55].

## 5. PI3K Inhibitors

The development of small molecule PI3K inhibitors aimed to block key signaling events that drive cancer cell survival [56]. The PI3K/AKT/mammalian target of the rapamycin (mTOR) pathway is frequently dysregulated in various tumors, making it an important target for drug development [57,58]. Various PI3K inhibitor types were developed and investigated in clinical trials, including pan-PI3K inhibitors, isoform-specific PI3K inhibitors, and dual PI3K/mTOR inhibitors.

Pan-PI3K inhibitors block all four PI3K isoforms (α, β, γ, and δ) and include buparlisib (BKM120), pictilisib (GDC-0941), and copanlisib [59]. Copanlisib received accelerated FDA approval for adult-relapsed FL in 2017 and was voluntary withdrawn from the market in 2024 because the required post-marketing trial did not verify the clinical benefit of copanlisib in FL. Both buparlisib and pictilisib demonstrated activity in solid tumors only, including glioblastoma, breast cancer, and ovarian cancer. Hyperglycemia is a common adverse effect of pan-PI3K inhibitors due to the blocking of the PI3K/AKT pathway, which is crucial for insulin receptor signaling.

Isoform-specific inhibitors target specific isoforms with improved efficiency and fewer adverse effects compared to pan-PI3K inhibitors. The PI3K-α inhibitors alpelisib and inavolisib are both FDA-approved for PIK3CA-related overgrowth spectrum and breast cancer, respectively [60,61]. The PI3K-β inhibitor AZD8186 was investigated in PTEN-deficient breast and prostate cancer because PTEN-deficient cancers often rely on the PI3Kβ pathway for survival, making it a viable therapeutic target [62]. On the other hand, PI3K-δ inhibitors were mainly targeted for hematologic malignancies. Idelalisib was FDA-approved in 2014 for relapsed FL and SLL. However, due to its severe toxicities such as colitis, hepatitis, pneumonitis, and infection, idelalisib was withdrawn in 2022 [63]. Similarly, umbralisib was initially FDA-approved in 2021 for relapsed/refractory MZL and FL, but was withdrawn in 2022 due to severe adverse effects [64]. Duvelisib is a dual isoform PI3K-δ/γ inhibitor with strong delta activity and is FDA-approved for relapsed or refractory CLL and SLL [65]. Duvelisib is not recommended as initial or second-line treatment due to an increased risk of treatment-related mortality. PI3K-γ inhibitors include eganelisib (IPI-549), which demonstrated activity in a subset of AML cells that showed dependency on the PI3K-γ signaling [66].

Dual PI3K/mTOR inhibitors target both PI3Ks and mTOR and include dactolisib, gedatolisib, paxalisib, and voxtalisib. Dactolisib toxicities hampered its development in any type of blood cancers. Gedatolisib did not show any clinical benefit in relapsed or refractory AML but demonstrated efficacy in HR+/HER2-advanced breast cancer. Paxalisib was also investigated in solid tumors and lymphomas. Preliminary results from a Phase 2 clinical trial demonstrated clinical activity of paxalisib in recurrent or refractory primary central nervous system lymphoma, including partial responses and stable disease (NCT04906096). Voxtalisib was evaluated for safety and efficacy in relapsed or refractory lymphoma, CLL, and SLL. Voxtalisib demonstrated an acceptable safety profile and efficacy against FL and limited efficacy against MCL, CLL, or SLL [67].

## 6. BCL-2 Inhibitors

The BCL-2 protein family is composed of both anti-apoptotic and pro-apoptotic proteins that guide apoptotic cell death. The main conserved motifs in the BCL2 family are the Bcl-2 homology (BH) domains, which include BH1, BH2, BH3, and BH4. These motifs are crucial for the family’s function in regulating apoptosis. Anti-apoptotic proteins, such as BCL-2, contain all four domains, whereas the pro-apoptotic members include the multidomain effectors (BAX and BAK, which contain BH1–3 domains) and the BH3-only proteins (BIK and BIM). The BH3 domain is responsible for Bax/Bak oligomerization that triggers mitochondrial rupture, cytochrome c release, and apoptosis. BCL-2 inhibits this process by binding to BAX at the BH3 domain and preventing further downstream apoptosis [68].

Venetoclax is a BH3 mimetic and the only FDA-approved BCL-2 inhibitor for treatment of CLL, SLL, and AML in combination with DNA methyltransferase inhibitors or low-dose cytarabine [69]. Tumor lysis syndrome, neutropenia, and infections are possible adverse effects [70]. Resistance to venetoclax occurred through the upregulation of anti-apoptotic proteins, as well as through mutations or modifications within the BCL-2 binding domains [71,72].

In the setting of relapsed or refractory CLL or AML, combination therapies involving venetoclax can be used to increase response and overcome resistance. These combinations exploit distinct but complementary mechanisms of action. For instance, BCL-2 inhibition sensitized leukemic cells to apoptosis, while BTK inhibitors and anti-CD 20 antibodies disrupted survival signaling pathways. Accordingly, one of the most widely utilized combinations is venetoclax with anti-CD20 monoclonal antibodies (mAbs) like obinutuzumab or rituximab. Furthermore, the combination of ventoclax and BTK inhibitors demonstrated success in clinical trials [73]. DNA hypomethylating agents such as azacitidine or decitabine were also combined with venetoclax in clinical settings, offering a secondary treatment option for patients with relapsed or refractory AML. Together, venetoclax and hypomethylating agents may serve as a bridge until stem cell transplantation, thus prolonging survival [74]. Triple therapy regimens involving BTK inhibitors, BCL-2 inhibitors, and anti-CD-20-antibodies demonstrated enhanced efficacy in preclinical studies compared to any single therapy alone. Triple therapy remains under evaluation in ongoing clinical trials [75].

New emerging BCL-2 inhibitors include LP-118 and navitoclax. Following treatment with venetoclax, patients who relapse often maintain sensitivity to BCL-2 inhibitors, making anti-apoptotic BCL-2 proteins an ongoing target for therapy. Targeting other members of the BCL-2 family like the anti-apoptotic B-cell lymphoma-extra-large (BCL-XL) could be an effective strategy to overcome resistance. LP-118 is a BCL-2/BCL-XL inhibitor that demonstrated efficacy in venetoclax-naive and venetoclax-resistant CLL, primarily through BAK activation and cytochrome C release. Its broader activity compared to venetoclax allowed it to be effective in cells harboring the BCL-2 G101V mutation, while also minimizing platelet toxicity [76]. Navitoclax is an orally bioavailable small molecule inhibitor of multiple anti-apoptotic proteins of the BCL-2 family including BCL-2, BCL-XL, and BCL-w [77]. Its clinical use is limited by dose-dependent adverse effects like thrombocytopenia and neutropenia resulting from BCL-XL inhibition, and by the availability of more selective agents like venetoclax [78].

## 7. Selective Inhibitors of Nuclear Export

Selective inhibitors of nuclear export (SINEs) are drugs that inhibit the nucleo-cytoplasmic transport of RNA and proteins by blocking the exportin-1 (XPO1) transporter. XPO1 is the nuclear exporter of several tumor suppressors and growth regulatory proteins like p21, p53, p73, FOXO, and STAT3 [79]. Overexpression and increased XPO1-mediated transport were observed in several types of solid and hematological tumors [80,81]. Accordingly, the inhibition of XPO1 is a rational approach to inhibit tumor growth.

Selinexor was the first orally active SINE to obtain FDA approval in 2019 for treatment of relapsed or refractory multiple myeloma (MM) and diffuse large B-cell lymphoma (DLBCL). Preclinical data demonstrated a synergistic effect of selinexor in combination with dexamethasone by blocking the nuclear export of phosphorylated glucocorticoid receptors and increasing their transcriptional activity [82]. Similarly, a synergistic effect was also observed with the proteasome inhibitor bortezomib in preclinical MM models. The synergistic activity was explained by the decreased activity of NF-kB through the increased stability of its inhibitor, IκB [83].

The triple combination of selinexor, dexamethasone, and bortezomib was also investigated in relapsed or refractory MM patients in a phase 1b/2 study [84]. The overall response rate was 43% and 84% for proteasome inhibitors-refractory and non-refractory patients, respectively. The triple combination is approved for MM patients who have received at least one prior therapy. Gastrointestinal toxicity including weight loss, neurological toxicity, thrombocytopenia, and neutropenia are possible adverse effects of selinexor.

## 8. Immune Therapies

### 8.1. Monoclonal Antibodies

mAbs were pivotal in the therapy of multiple hematologic malignancies, offering targeted strategies beyond traditional chemotherapy. mAbs are produced by identical B-lymphocyte clones and target specific antigen epitopes on tumor cells to induce tumor killing by mechanisms such as complement-dependent cytotoxicity (CDC), antibody-dependent cell-mediated cytotoxicity (ADCC), or direct induction of apoptosis. High specificity of these mAbs resulted in lower systemic toxicity and adverse effects compared to standard chemotherapy treatment [85]. Different types of anti-CD20 antibodies induced different patterns of CD20 redistribution and consequent activation of CDC, ADCC, and signal transduction pathways. Type I anti-CD20 mAbs, such as rituximab, redistributed CD20 molecules to lipid rafts microdomains on the cell membrane and were associated with strong CDC activity. On the other hand, type II anti-CD20 mAbs bound to different epitopes on CD20 outside lipid rafts with minimal internalization, no redistribution, and a greater amount of antibody remains on the surface. Type II anti-CD20 mAbs were developed to overcome type I resistance and was associated with potent B-cell depletion, enhanced direct cell death, and a weaker CDC [86,87]. Table 3 lists the different generations of mAbs and their target epitopes.

#### 8.1.1. First-Generation Anti-CD20: Rituximab

Rituximab, a chimeric monoclonal IgG1 antibody, was one of the first mAbs developed to treat CLL. Rituximab exerts its therapeutic effect by binding to the extracellular loop of CD20 and inducing B-cell lysis primarily through two key mechanisms: CDC and ADCC. CLL response rate was improved by the combination of Rituximab with chemotherapy [12]. Notable adverse effects include severe mucocutaneous reactions like paraneoplastic pemphigus and Stevens–Johnson syndrome [88].

**Table 3:** **The target epitopes of monoclonal antibodies used in hematologic malignancies.** INO indicates Inotuzumab ozogamicin, GO indicates Gemtuzumab ozogamicin, ADC indicates antibody drug conjugate, and N/A indicates not applicable.

Drug Name	Generation	Type	Target Epitope	References
Rituximab	First	I	CD20 small extracellular loop	[12]
Ofatumumab	Second	I	CD20 small and large extracellular loops	[89]
Ocrelizumab	Second	I	CD20 large extracellular loop	[90]
Veltuzumab	Second	I	CD20 large extracellular loop	[90]
Obinutuzumab	Third	II	CD20 large extracellular loop	[91]
Ublituximab	Third	I	CD20 large extracellular loop	[86]
Alemtuzumab	First	N/A	CD52	[92]
Epratuzumab	First	N/A	CD22	[93]
(INO)	First	N/A	CD22	[94]
(GO)	First	N/A	CD33	[95]
Tafasitamab	Second	N/A	CD19	[96]
Loncastuximab (ADC)	N/A	N/A	CD19	[97]
Daratumumab	First	N/A	CD38	[98]
Isatuximab	Next-generation	N/A	CD38	[98]
Brentuximab (ADC)	N/A	N/A	CD30	[99]
Elotuzumab	First	N/A	SLAMF7	[100]

#### 8.1.2. Second-Generation Anti-CD20: Ofatumumab, Ocrelizumab, and Veltuzumab

Ofatumumab is an anti-CD20 antibody composed of immunoglobulins derived from more than 90% human germline sequences, thereby reducing the risk of immunogenicity [101]. Ofatumumab is currently FDA-approved to treat CLL patients refractory to fludarabine and alemtuzumab [89]. Ofatumumab binds to both the small and large extracellular loop of CD20, resulting in tighter binding and increased efficacy [102]. Ocrelizumab is a humanized mAb studied in the past decade as a type I second-generation anti-CD20 antibody. Compared to rituximab, ocrelizumab showed improved efficacy against lymphoid cancers and stronger binding to the low-affinity variants of the FcγRIIIa (CD16) receptor, and consequently enhanced ADCC and reduced CDC [90]. An overall response rate of about 38% was achieved in a phase I/II clinical trial of patients with relapsed or refractory FL after failing prior rituximab therapy. The response was similar in patients with low-affinity and high-affinity variants of the FcγRIIIa [103]. Since B-cells are a key component of the immune attack in multiple sclerosis, ocrelizumab efficacy in the treatment of multiple sclerosis was studied. Ocrelizumab became the first FDA-approved drug for relapsing and primary progressive multiple sclerosis in 2017 [104].

Veltuzumab (formerly IMMU-106) is a type I humanized anti-CD20 mAbs, currently in early clinical development for lymphoid cancers and immune thrombocytopenia (ITP). It binds to a different epitope on the CD20 protein than rituximab, exhibiting enhanced ADCC and greater affinity for the FcγRIIIa receptor [90]. A phase I study of subcutaneous low-dose veltuzumab in previously untreated or relapsed CLL showed major leukemic cell reductions in all patients with a disease control rate of 83% [105]. As of 2015, the only U.S. regulatory designation for veltuzumab is an orphan drug designation for immune thrombocytopenia [106].

#### 8.1.3. Third-Generation Anti-CD20: Obinutuzumab and Ublituximab

Obinutuzumab is the newest type II humanized anti-CD20 antibody with a glycoengineered Fc portion that enhances the binding affinity of effector cells to the FcγRIII receptor [91]. While obinutuzumab’s mechanism of killing lacks CDC, it mediates B-cell lysis through improved ADCC and direct cell death [107]. Obinutuzumab showed promise in extending progression-free survival in CLL patients [108]. Ublituximab (TG-1101) is a chimeric, glycoengineered antibody under evaluation in phase III clinical trials for patients with hematologic malignancies [86]. Early phase I/II trials achieved an overall 43% response rate in rituximab relapsed or refractory patients with B-cell non-Hodgkin lymphoma (NHL) or CLL [109]. Another phase II study combined ublituximab and umbralisib (U2 combo) with ibrutinib for CLL patients with detectable minimal residual disease (MRD); results showed that 52% achieved undetectable MRD, and 63% entered treatment-free observation [102].

#### 8.1.4. Anti-CD52 Antibodies

Alemtuzumab is a humanized IgG1 mAb that binds to CD52 on neutrophils, B-cells, and T-cells. It induces cell death through ADCC and CDC [12]. It is approved as monotherapy for B-cell CLL, where it reduces the number of B-cells in blood and bone marrow [92]. Its adverse effects include myelosuppression and immunosuppression due to depletion of immune cells, so prophylaxis against infection with *Pneumocystis carinii* and herpesvirus is highly recommended [12]. Following treatment, the reconstitution of immune cells induced the emergence of clonal CD52- and CD8+ T-cells that were associated with long-lasting disease remission in patients with CLL [92]. While most CLL patients achieved partial remission [110], alemetuzumab was shown to be particularly beneficial for those who were resistant to chemotherapy treatment, as it preferentially cleared malignant cells from the blood [111]. Unfortunately, alemtuzumab monotherapy is insufficient for long term remissions of CLL [112]. Similarly to ocrelizumab, it is used in the treatment of relapsing forms of multiple sclerosis by depleting both the B-cells and T-cells.

#### 8.1.5. Anti-CD22 Antibodies

Epratuzumab is a humanized IgG1 naked anti-CD22 mAb evaluated in phase I/II trials for B-cell precursor ALL, especially for children. Many studies showed modest activity and demonstrated limited efficacy compared with standard treatment and alternative therapies [93,113].

Inotuzumab ozogamicin (INO) is an anti-CD22 antibody drug conjugate (ADC)—a humanized anti-CD22 IgG4 antibody linked to calicheamicin, a cytotoxic antibiotic. After binding to CD22 by surface lysine residues, it is rapidly internalized, releasing calicheamicin by hydrolytic cleavage, and inducing double-strand DNA breaks and apoptosis. Studies demonstrated its activity as monotherapy and in combination with rituximab or chemotherapy. Subsequent studies with INO in combination therapy with the bispecific antibody blinatumomab and others were also investigated. It is currently used for adults with relapsed or refractory CD22-positive B-cell ALL [114]. The INO-VATE phase III trial compared the outcomes of INO and standard chemotherapy in relapsed or refractory ALL and showed that INO resulted in higher rates of complete remission and complete remission with incomplete hematologic recovery [94].

#### 8.1.6. Anti-CD19 Antibodies

CD19 is a transmembrane glycoprotein expressed on B-cells throughout their development and serves as a biomarker for B-cell-related disorders. Targeting CD19 using mAbs or bispecific antibodies is a rational strategy because of its physiological role in B-cell development from their early differentiation events in the bone marrow to late maturation steps in the spleen [115]. Tafasitamab is an Fc-modified mAb that binds to CD19 antigen expressed on the surface of several B-cell tumors, including DLBCL and FL. Upon binding to CD19, it mediates B-cell lysis through ADCC and antibody-dependent cellular phagocytosis (ADCP). In vitro lymphoma model studies using the combination of tafasitamab and lenalidomide demonstrated increased ADCC activity compared to tafasitamab or lenalidomide alone [116]. Tafasitamab was FDA-approved in 2020 and indicated for relapsed or refractory DLBCL in combination with lenalidomide. Recently, it was also approved for treatment of relapsed or refractory FL in combination with lenalidomide and the anti-CD20 rituximab.

Loncastuximab is another CD19 mAb that was conjugated to the alkylating agent tesirine to form an ADC. Upon binding to CD19, the ADC is internalized followed by release of the alkylating agent, which binds to the DNA minor groove and forms highly cytotoxic DNA interstrand crosslinks. Loncastuximab is indicated for relapsed or refractory large B-cell lymphoma (LBCL) after two or more lines of systemic therapy. Myelosuppression and infections are possible adverse reactions for both tafasitamab and loncastuximab.

#### 8.1.7. Anti-CD33 Antibodies

Gemtuzumab ozogamicin (GO) is a humanized IgG4 anti-CD33 ADC used to target AML cells. Similarly to INO, it is also linked to calicheamicin and induces DNA strand breaks, leading to mitochondrial apoptosis [95]. Approved by the FDA in 2017, GO is indicated for patients with CD33-positive AML based on a phase III ALFA-0701 trial, which demonstrated that a reduced dose of GO with standard chemotherapy improved survival in adults with AML [117]. Other anti-CD33 antibodies showed suboptimal results in different studies. A phase III trial found that combining lintuzumab (SGN-33) with chemotherapy was safe with minimal toxicity but did not significantly improve remission rates or overall survival over standard chemotherapy [118]. Phase I studies of SGN-CD33A (vadastuximab talirine) demonstrated promising activity and remission rates when combined with chemotherapy; however, concerns with liver and hematopoietic cell toxicity led to termination of further trials [119].

#### 8.1.8. Anti-CD38 Antibodies

Daratumumab was the first anti-CD38 mAb approved in 2015 for relapsed or refractory MM [120]. It was also approved for newly diagnosed MM in triple or quadruple combination therapy. Triple therapy included D-Rd (daratumumab, lenalidomide, and dexamethasone) from the MAIA trial and D-VMP (daratumumab, bortezomib, melphalan, and prednisone) from the ALCYONE trial [121,122]. Quadruple therapy included D-VRd (daratumumab, bortezomib, lenalidomide, and dexamethasone) from the PERSEUS trial and D-VTD (daratumumab, bortezomib, thalidomide, and dexamethasone) from the CASSIOPEIA trial in Europe [123,124]. Daratumumab’s adverse effects included hematologic toxicity, respiratory tract infections, fatigue, and gastrointestinal symptoms [125].

Isatuximab is another IgG1 anti-CD38 mAb approved in 2020 with similar mechanism as daratumumab, but it also induced apoptosis directly without cross-linking by CD8+ T and NK cell [126]. Approved combination agents include Isa-Pd (isatuximab, pomalidomide, and dexamethasone) and Isa-Kd (isatuximab, carfilzomib, and dexamethasone) [127,128]. A phase III trial is investigating Isa-VRd (isatuximab, bortezomib, lenalidomide, and dexamethasone) in patients with newly diagnosed MM [129]. The safety profile of isatuximab was similar to daratumumab, with emphasis on infusion-related reaction, cytopenias, and infections [127].

#### 8.1.9. Anti-CD30 Antibodies

CD30 (also known as TNFRSF8) is a member of the tumor necrosis factor (TNF) receptor superfamily. The endogenous ligand for CD30 is a membrane bound cytokine with sequence homology to other members of the TNF family. CD30 is expressed in a variety of lymphoid neoplasms with the highest expression in classical Hodgkin lymphoma (cHL) and anaplastic large cell lymphomas (ALCL) [99]. This observation suggested a role for CD30 in the development and progression of cancer in these lymphomas. Preclinical studies demonstrated efficacy of murine anti-CD30 mAbs in mice xenograft models and ALCL cell lines [130,131]. Brentuximab vedotin was the first FDA-approved ADC targeting CD30 and consisted of a chimeric IgG1 mAb conjugated to the microtubule-disrupting agent monomethylauristatin E (MMAE). Brentuximab induced cell cycle arrest and apoptosis in tumor cells through binding to CD30, internalization, and release of MMAE that disrupts the microtubule network. It is indicated for cHL and systemic ALCL and recently approved for LBCL. Peripheral neuropathy (probably mediated through the anti-tubulin effect of MMAE), febrile neutropenia, infections and opportunistic infections, progressive multifocal leukoencephalopathy (PML), and hepatic and pulmonary toxicities were reported as adverse effects after administration of brentuximab. Loss of CD30 expression was reported in cases of ALCL after treatment with brentuximab with consequent resistance development [132,133]. Other mechanisms of resistance development like the upregulation of drug efflux transporters and MMAE resistance were also reported [134].

#### 8.1.10. Anti-SLAMF7 Antibodies

SLAMF7 (also known as CD319) is a cell membrane glycoprotein highly expressed in malignant plasma cells. It is also expressed in NK cells, T-cells, B-cells, dendritic cells and monocytes. It is a member of the SLAM family of receptors and plays a role in lymphocytes’ survival and development [135].

Elotuzumab is a humanized IgG1 mAb that specifically targets SLAMF7 and interacts with Fc receptors on effector NK cells to mediate the killing of myeloma cells through ADCC. An interesting study investigated the role of elotuzumab on other effector cells in the tumor microenvironment using a tumor xenograft model deficient in NK and adaptive immune cells. Elotuzumab demonstrated antitumor activity in the xenograft model through the activation of tumor-associated macrophages (TAMs) and consequent ADCP of myeloma cells [100]. These data showed that elotuzumab could induce myeloma cells cytotoxicity through both ADCC and ADCP. The combination of elotuzumab and lenalidomide in preclinical models resulted in enhanced activation of NK cells compared to single agents and increased antitumor activity in vitro and in vivo [136]. Elotuzumab is indicated in combination with lenalidomide and dexamethasone for the treatment of MM adult patients who have received one to three prior therapies. Infections, infusion reactions, and second primary malignancies were reported as adverse effects with the use of elotuzumab.

### 8.2. Radioimmunoconjugates

Radioimmunoconjugates (RICs) are mAbs labeled with radionuclides to deliver targeted radiation by binding to a specific antigen on tumor cells, thereby combining immune targeting with radiation therapy in a single agent. After the mAb binds to the antigen, the attached radioisotope emits radiation to kill the targeted cell and potentially nearby cells. RICs are used in AML and currently being evaluated for relapsed/refractory ALL and AML. Leukemia-associated antigens include CD22, CD33, CD45, CD66, and others [137]. Epratuzumab tetraxetan and BAY1862864 are RICs that eliminate B-cells through distinct mechanisms, where epratuzumab tetraxetan causes cell death by beta decay, while BAY1862864 emits alpha particles, causing cell cycle arrest via DNA damage [138]. Epratuzumab tetraxetan showed a 62% overall response rate (ORR), 48% complete response rate (CRR), and median progression-free survival (PR) of 9.5 months in 64 patients with relapsed or refractory non-Hodgkin lymphoma (NHL) [139]. The combination of epratuzumab tetraxetan with veltuzumab was tested in 18 patients with aggressive NHL; the ORR was 53% with 13 patients experiencing grade 3–4 neutropenia or thrombocytopenia [140]. HuM195 is a humanized anti-CD33 mAb that could be labeled with either alpha-emitters or beta-emitters. In a previous study with 18 relapsed or refractory AML or CML patients, HuM195 was conjugated with an alpha-emitting isotope. Most patients had reductions in both circulating and bone marrow blasts with no significant extramedullary toxicity [141]. Another phase III study in relapsed or refractory AML patients utilized an anti-CD45 RIC called Iomab-B (I-131 apamistamab). Results showed higher complete remission with a favorable safety profile and lower rates of sepsis compared to patients who underwent standard-of-care treatment due to the targeted approach [142].

### 8.3. Chimeric Antigen Receptor T-Cells

Chimeric Antigen Receptor (CAR) T-cells are lymphocytes—most commonly T-cells —that have been genetically engineered to recognize a leukemia-specific antigen for cell elimination. CAR binding to specific antigens on the cell surface is independent of MHC receptor-induced T-cell activation. Anti-CD19 CAR T-cell therapy is FDA-approved for B-cell precursor acute lymphoblastic leukemia (B-ALL) and under investigation for the treatment of AML with antigen targets such as CD33 and CD123. Unfortunately, CAR T-cell therapy is associated with serious adverse effects like cytokine release syndrome (CRS), which could be life-threatening. Secondary T-cells malignancies were also reported following treatment of hematologic malignancies with BMCA- and CD19-directed CAR T-cell therapies [143].

Antigen escape-related relapse is another drawback of CAR T-cell therapy and occurs when leukemia cells lose or mutate the target antigen [143,144]. In a phase I/II study of the anti-CD19 CAR T-cell therapy tisagenlecleucel in 75 pediatric and young adult patients with CD19+ relapsed/refractory ALL (ELIANA trial), the overall remission rate within 3 months was 81%, with event-free survival and overall survival of 73% and 90% at 6 months and 50% and 76% at 12 months, respectively. CRS occurred in 77% of patients and neurologic events occurred in 40% of patients. The study concluded that tisagenlecleucel provided adequate remission rates but resulted in high-grade toxicity [145]. Another phase II study (ZUMA-3) evaluated CAR T-cell therapy KTE-X19 in patients of similar criteria with relapsed/refractory B-cell ALL. It was effective and achieved deep and durable remissions with a manageable safety profile [146]. For CD33 CAR T-cell therapy, a phase I trial (NCT03126864) assessed its safety and effectiveness in ten relapsed/refractory AML patients. The results demonstrated its efficacy only in patients with higher lymphocyte count and lower blood blasts [147]. Another study addressed antigen escape relapse drawback by the development of CD123/B7-H3 bispecific universal CAR-T (UCAR-T) cells using CRISPR gene editing technology. Results in AML xenograft models showed inhibition of tumor progression, extended survival, and absence of CRS [148]. CAR T-cell therapy targeting B-cell maturation antigen (BCMA) in MM patients also demonstrated efficacy. Several BCMA-targeted CAR T-cell therapies were under clinical development for patients with relapsed or refractory MM. The KarMMa-2 trial investigated idecabtagene vicleucel (Ide-cel), which targeted BCMA, and responses were observed in nearly three-fourths of patients with heavily pretreated, highly relapsed, or refractory MM [149]. Ide-cel was FDA-approved in 2021 and indicated for relapsed or refractory MM patients after two or more prior lines of therapy. Ciltacabtagene autoleucel was another BCMA-targeted CAR T-cell therapy that received approval in 2022 and was indicated for patients with relapsed or refractory MM who were refractory to lenalidomide and had received at least one prior line of therapy. Table 4 summarizes the FDA-approved CAR T-cell therapies indicated for various types of blood cancer.

**Table 4:** **List of CAR T-cell medications approved for different types of blood cancer.** B-ALL: B-cell precursor acute lymphoblastic leukemia; LBCL: large B-cell lymphoma; CLL: chronic lymphocytic leukemia; SLL: small lymphocytic lymphoma; FL: follicular lymphoma; MCL: mantle cell lymphoma; MM: multiple myeloma; R/R: relapsed or refractory; R1: refractory to first-line chemoimmunotherapy; 2L: patients who had received at least two prior lines of therapy; and 1L: patients who had received at least one prior line of therapy.

Generic Name (Brand)	Target	Indications	References
Tisagenlecleucel (Kymriah)	CD19	R/R B-ALL R/R LBCL (2L) R/R FL (2L)	[145]
Brexucabtagene autoleucel (Tecartus)	CD19	R/R MCL R/R B-ALL	[146]
Obecabtagene autoleucel (Aucatzyl)	CD19	R/R B-ALL	[150]
Lisocabtagene maraleucel (Breyanzi)	CD19	R/R LBCL (R1) R/R CLL or SLL (2L) R/R FL (2L) R/R MCL (2L)	[151]
Axicabtagene ciloleucel (Yescarta)	CD19	R/R LBCL (R1)	[152]
Idecabtagene vicleucel (Abecma)	BCMA	R/R MM (2L)	[153]
Ciltacabtagene autoleucel (Carvykti)	BCMA	R/R MM (1L)	[154]

### 8.4. Immune Checkpoint Inhibitors

Immune checkpoints are proteins that prevent the immune system from attacking tumor cells. Inhibition of these checkpoint proteins stimulated the immune system to attack tumor cells. CTLA-4, PD-1, PD-L1, and LAG-3 are examples of these checkpoint proteins and several mAbs were developed to inhibit them like ipilimumab, pembrolizumab, nivolumab, atezolizumab, and relatlimab [155]. These drugs probably demonstrated more efficacy in solid tumors compared to hematologic malignancies because of the higher immune cell infiltration into the tumor microenvironment [156].

**Figure 2:**
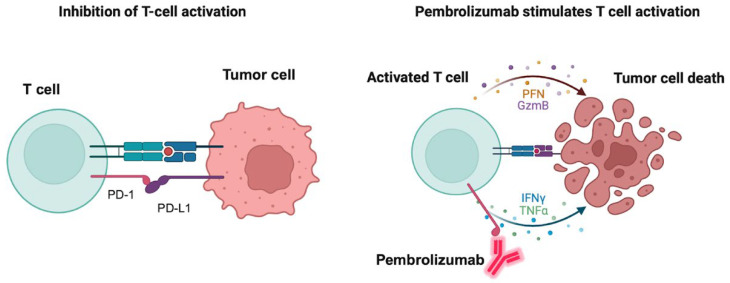
**Mechanism of action of pembrolizumab.** The left panel shows the binding of the immune checkpoint-programmed death receptor 1 (PD-1) to its endogenous ligand PD-L1 with consequent inhibition of T-cell activation and prevention of tumor cells attack. The right panel shows the binding of pembrolizumab to PD-1 with consequent activation of T-cells and release of the apoptosis mediators granzyme B (GzmB), perforin (PFN), tumor necrosis factor (TNF) alpha and interferon (IFN) gamma with consequent tumor cell apoptosis. Created in BioRender. Fandy, T. (2025) https://BioRender.com/y8s65re (accessed on 27 October 2025).

Pembrolizumab is an FDA-approved PD-1 inhibitor for treatment of hematologic tumors like cHL and primary mediastinal LBCL. It is also approved for the treatment of a variety of solid tumors including non-small cell lung cancer, melanoma, gastric and esophageal cancer, HCC, RCC, and triple-negative breast cancer. Severe or fatal immune-mediated inflammatory adverse reactions could occur in the lungs, colon, liver, kidney, endocrine glands or the skin, which may require the withholding or discontinuation of pembrolizumab. Hypothyroidism may also be observed because of immune-mediated thyroiditis. Figure 2 depicts the mechanism of action of the PD-1 inhibitor pembrolizumab and how it induces tumor cell death. Nivolumab is another PD-1 inhibitor that is also indicated for relapsed or refractory cHL and shares similar adverse effects with pembrolizumab.

### 8.5. Bispecific Antibodies (T-Cell Engagers)

T-cell engagers (TCEs) are bispecific antibodies capable of simultaneous binding to two different antigens. One antigen is expressed on the surface of the tumor cell and the other antigen is the CD3 surface marker expressed on T-cells. When the TCE binds to both antigens, it brings them close together and allowing the T-cells to recognize and kill the tumor cells.

#### 8.5.1. TCEs Targeting CD19

Blinatumomab is a bispecific antibody composed of variable light (VL) and variable heavy (VH) domains derived from anti-CD3 and anti-CD19 IgG molecules [12]. Because ALL blast cells frequently express CD19, they became an ideal target for both mAbs and TCEs [157]. Blinatumomab functioned by generating a synapse between cytotoxic T-cells expressing CD3 and ALL cells expressing CD19, thus enabling T-cells to recognize and lyse malignant B-cells [12]. Early clinical trials with blinatumomab demonstrated high response rates with durable remissions, highlighting the ability of T-cells to efficiently and sustainably eliminate malignant cells [158]. Compared with rituximab, blinatumomab required lower concentrations to achieve similar cytotoxicity. While both agents activated pro-caspases 3 and 7 and induced granzyme-mediated apoptosis, their combination further enhanced therapeutic efficacy, particularly at low effector-to-target cell ratios [159].

Blinatumomab is FDA-approved for relapsed or refractory B-ALL and MRD-positive B-ALL. It also demonstrated cytotoxicity against chemotherapy-resistant cells that could lead to hematologic relapse [160]. MRD testing via PCR or flow cytometry detected the presence of these resistant cells and provided a rationale for using blinatumomab [161]. Subsequent studies demonstrated the efficacy of blinatumomab in treating both Ph+ and Ph- B-ALL across age groups [162]. Common adverse effects included CRS, neutropenic fever, sepsis due to immunosuppression, and neurotoxicity [12].

#### 8.5.2. TCEs Targeting GPRC5D

GPRC5D is a transmembrane protein that acted as a receptor for unknown ligands. It is highly expressed in MM cells compared with normal plasma cells and plays a role in cellular proliferation and resistance to chemotherapy. Talquetamab is a bispecific GPRC5D-directed TCE that showed potent antitumor activity in MM bone marrow samples, including those with high-risk cytogenetic aberrations [163].

The efficacy of talquetamab depended on the expression level of GPRC5D and the effector-to-target (E:T) ratio, where high expression level of GPRC5D and a high E:T ratio were associated with improved lysis of MM cells. Conversely, an increased proportion of T-cells expressing PD-1 or HLA-DR and elevated regulatory T-cell (Treg) counts were associated with decreased cytotoxicity [163]. Talquetamab is indicated for the treatment of adult patients with relapsed or refractory MM who had received at least four prior lines of therapy, including a proteasome inhibitor, an immunomodulatory agent, and an anti-CD38 mAb. Reported adverse reactions included cytopenias, CRS, neurologic toxicity, weight loss, and respiratory infections.

#### 8.5.3. TCEs Targeting B-Cell Maturation Antigen

BCMA is a cell membrane receptor expressed on the surface of plasma cells and overexpressed in MM cells. BCMA plays a role in the survival and growth of plasma cells by interacting with ligands such as BAFF and APRIL. High levels of soluble BCMA in the plasma were associated with poorer clinical outcomes in MM patients [164]. Accordingly, BCMA was considered a rational therapeutic target for immunotherapies utilizing bispecific BCMA-directed TCEs such as teclistamab, elrenatamab, and linvoseltamab. The three drugs were indicated for the treatment of adult patients with relapsed or refractory MM who had received at least four prior lines of therapy, including a proteasome inhibitor, an immunomodulatory agent, and an anti-CD38 mAb [165,166]. Reported adverse reactions included CRS, neurologic toxicity, cytopenias, and upper respiratory tract infections.

#### 8.5.4. TCEs Targeting CD20

Glofitamab, epcoritamab, and mosunetuzumab are CD20-directed TCEs indicated for adult patients with relapsed or refractory B-cell NHL after two or more lines of systemic therapy. In vitro studies demonstrated that these agents activated T-cells, triggered the release of proinflammatory cytokines, and induced lysis of B-cells [167]. Reported adverse reactions were similar to those observed with other TCEs.

### 8.6. Immunomodulatory Drugs (IMiDs)

IMiDs are thalidomide analogs used in treatment of MM and specific types of NHL. Thalidomide, lenalidomide, and pomalidomide are FDA-approved for treatment of MM in combination with dexamethasone. Lenalidomide is also indicated for MCL as a single agent or in combination with rituximab for treatment of previously treated FL and MZL. The antitumor mechanism of IMiDs is still not fully understood, but their antiproliferative, anti-inflammatory, antiangiogenic, and immune modulation effects are believed to contribute to their clinical efficacy [168]. Antithrombotic prophylaxis is recommended with IMiDs therapy due to the increased risk of deep vein thrombosis and pulmonary embolism.

## 9. Proteasome-Dependent Drugs

The proteasome is a multiprotein protease complex responsible for degrading ubiquitinated cellular proteins. Proteasome inhibitors demonstrated antitumor activity across multiple tumor types by disrupting signaling cascades and inducing cell death [169]. The first-generation reversible proteasome inhibitor bortezomib was FDA-approved in 2003 for the treatment of MM. Bortezomib demonstrated efficacy in both newly diagnosed and relapsed or refractory MM patients; however, the development of resistance and adverse effects such as peripheral neuropathy were major drawbacks that often led to therapy discontinuation. Bortezomib was also approved for the treatment of MCL, either as a single agent or in combination with chemotherapy [170].

The second-generation irreversible proteasome inhibitor carfilzomib was developed to overcome the limitations of bortezomib. Preclinical MM models demonstrated that carfilzomib induced apoptosis in bortezomib resistant MM cell lines and patient-derived MM cells. The combination of carfilzomib with dexamethasone synergistically reduced the proliferation of MM cells [171]. Cardiovascular adverse events such as heart failure, ischemia, arrhythmias, and hypertensive crises were reported with the use of carfilzomib [172]. Ixazomib is another second-generation proteasome inhibitor prodrug used in combination with lenalidomide and dexamethasone for MM patients who had received at least one prior therapy. Ixazomib induced apoptosis in MM cell lines and in primary cells from MM patients who had relapsed after bortezomib therapy. It also demonstrated antitumor activity in a mouse MM xenograft model [173]. Ixazomib is not recommended for use in newly diagnosed or maintenance therapy for MM patients. Similarly to bortezomib, peripheral neuropathy was reported as an adverse effect, in addition to thrombocytopenia. Several clinical trials reported the efficacy of bortezomib-based combination therapy in other hematologic malignancies, including T-cell lymphomas [174], AML [175], ALL [176], and indolent B-cell NHL [170].

**Figure 3:**
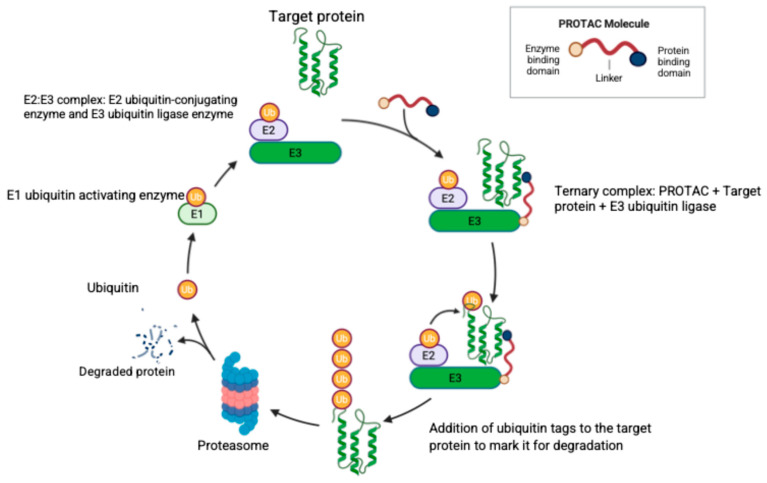
**The structure of a PROTAC molecule and its mechanism of specific protein degradation.** The PROTAC molecule consists of three domains as illustrated, where the linker domain joins the target protein binding domain and the E3 ubiquitin ligase binding domain. Ubiquitin (Ub) is activated by the Ub-activating enzyme (E1) and then transferred to the complex (E2–E3) formed by the Ub-conjugating enzyme (E2) and the Ub-ligase enzyme (E3). The PROTAC molecule joins the target protein with the E2:E3 complex to form the ternary complex with consequent Ub tagging of the target protein and proteasomal degradation. Created in BioRender. Fandy, T. (2025) https://BioRender.com/1c1bcpu (accessed on 27 October 2025).

Another approach for targeting the proteasome is through the use of PROTACs. Targeted protein degradation via PROTACs is a promising new strategy in the treatment of both hematologic malignancies and solid tumors. PROTACs are bifunctional molecules joined by a linker to recruit E3 ubiquitin ligases to tag the target protein for proteasomal degradation [177]. Figure 3 illustrates the structure and mechanism of action of PROTACs. Currently, there are no FDA-approved PROTACs, but vepdegestrant (an estrogen receptor degrader for treatment of breast cancer) received the FDA fast track designation and is expected to obtain approval in a few years.

Early studies with mutated FLT3-ITD degraders showed significant inhibition of FLT3-ITD-positive cells growth, as well as downregulation of the phosphorylation of FLT3 and STAT5, which are key downstream molecules involved in AML pathogenesis [178]. Preclinical studies demonstrated that incorporating FLT3 inhibitors—such as quizartinib, dovitinib, and gilteritinib—as ligands into PROTACs enhanced both specificity and anti-leukemic efficacy. For instance, quizartinib incorporation into a PROTAC inhibited cell growth more potently than when it was used as a single agent. Additionally, the PROTAC induced higher levels of apoptosis, despite a slight reduction in the kinase inhibitory activity of the PROTAC compared to quizartinib, suggesting that FLT3-ITD could contribute to disease progression independent of its kinase activity [179]. Similarly, dovitinib was incorporated into a CRBN-recruiting PROTAC targeting mutated FLT3 and demonstrated strong antiproliferative activity [180]. Gilteritinib-based PROTACs, such as B3-2 and Z29, exhibited strong antitumor activity and B3-2 was effective against FLT3 resistant mutations [181]. Z29 demonstrated improved specificity compared to gilteritinib as a single agent and when combined with venetoclax, Z29 showed an improved safety profile with lower platelet and hepatic toxicities [182]. Another study designed a novel FLT3 gilteritinib PROTAC that also mediated the degradation of the transcription factor IKZF1/3 and GSPT1 through the use of molecular glue degraders (single small molecules that bind to the target proteins GSPT1 and IKZF1/3 and create a conformational change that promotes a non-canonical protein–protein interaction and stabilization between the target protein and E3 ubiquitin ligase). The novel multitarget PROTAC demonstrated improved antiproliferative activity against AML compared to gilteritinib [183]. Furthermore, another novel oral PROTAC (A20) targeting FLT3-ITD demonstrated complete tumor regression and improved survival in AML xenograft models. A20 showed complete elimination of CD45+CD33+ cells, which are commonly expressed on AML blasts [184].

GMB-475 is also a PROTAC that allosterically targeted the BCR-ABL protein in CML cells. GMB-475 decreased the viability and induced apoptosis in primary CML CD34^+^ cells without affecting the viability of healthy CD34^+^ cells [185]. DT2216 is another PROTAC that targeted the anti-apoptotic protein BCL-XL in T-ALL cells and inhibited the growth of several xenograft tumors, either as a single agent or in combination with other chemotherapeutic agents [186]. Since BTK played a key role in the survival of B-cell malignancies, PROTACs targeting BTK were considered a potential treatment strategy for B-cell lymphomas. NX-5948 is a selective BTK degrader that demonstrated efficacy in a xenograft mouse model containing the BTK C481S mutation and induced superior tumor growth inhibition compared with ibrutinib. TD-004 is also a PROTAC that targeted ALK and inhibited the growth of ALK fusion-positive lymphoma cell lines [187].

The development of PROTACs for the treatment of MM remained an active area of research. Targeting proteins such as PRMT4 [188], RAD51 [189], HDAC6 [190], and FKBP12 [191] for degradation demonstrated efficacy in preclinical in vitro and in vivo models. Although targeted protein degradation using PROTACs is a promising therapeutic strategy due to their ability to overcome resistance mechanisms, challenges that limited their clinical utility continued to emerge because of their poor drug-likeness, limited absorption, low stability, and insufficient specificity [192].

## 10. Conclusions

Molecularly targeted therapies offered effective alternatives for the standard chemotherapy treatment in different types of hematologic malignancies. The recent advances in identifying driver mutations and the classification of leukemias and lymphomas into distinct molecular subgroups created opportunities for the discovery and development of novel therapeutic entities. Over the past decade, the FDA-approved 29 new molecularly targeted therapies and 21 new immune therapies to treat various types of hematologic malignancies. These drugs significantly decreased blood cancer-related deaths and improved survival rates. For instance, mortality rates for NHL have declined by 47% between 1997 and 2023 [193].

Targeted therapy transformed cancer therapy by enabling personalized treatment of tumors evolved by specific mutations. However, the prediction of the clinical efficacy of some classes of targeted therapy in specific types of tumors remains a challenge. For example, it is not fully understood why BCl-2 inhibitors or proteasome inhibitors are not effective in most types of blood cancer. Moreover, some of the PI3K inhibitors and immune check inhibitors demonstrated higher efficacy in solid tumors compared to hematologic malignancies. The off-target adverse effects, resistance prediction and development are other major issues in these types of therapy. However, the development of newer generations of TKIs or FLT3 inhibitors, the use of isoform-specific inhibitors of PI3K, the use of mAbs, and PROTACs were successful approaches to enhance specificity and overcome resistance development against targeted therapy. Another approach to overcome drug resistance and reduce drug toxicity is the use of combination therapy. Nevertheless, the complexity of designing effective combinations is a significant challenge that could be overcome by the use of artificial intelligence to select drugs from different classes and predict their combination efficacy. Further research into potential strategies to overcome resistance mechanisms and to develop biomarkers will further optimize the clinical use of these drugs and enhance their clinical outcomes.

## Figures and Tables

**Table 2:** **Types of FLT3 inhibitors and their potency in inhibiting FLT3.** IC_50_ indicates the half-maximal inhibitory concentration expressed in nanomolar (nM).

Drug Name	Generation	Type	Potency (IC_50_) in nM
Lestaurtinib	First	I	3
Tandutinib	First	II	220
Midostaurin	First	I	6.3
Sorafenib	First	II	58
Sunitinib	First	I	7
Quizartinib	Second	II	1.6
Gilteritinib	Second	I	0.29
Crenolanib	Second	I	1.3

## Data Availability

No new data were created or analyzed in this study. Data sharing is not applicable to this article.

## Abbreviations

ADC	antibody drug conjugate
ADCC	antibody-dependent cell-mediated cytotoxicity
ADCP	antibody-dependent cellular phagocytosis
AKT	protein kinase B
ALCL	anaplastic large cell lymphomas
ALK	anaplastic lymphoma kinase
ALL	acute lymphoblastic leukemia
AML	acute myeloid leukemia
ATP	adenosine triphosphate
B-ALL	B-cell precursor acute lymphoblastic leukemia
BCMA	B-cell maturation antigen
Bcl-XL	B-cell lymphoma-extra large
BCR	B-cell receptor
BCR-ABL	breakpoint cluster region-Abelson
BTK	Bruton tyrosine kinase
CAR	chimeric antigen receptor
CDC	complement-dependent cytotoxicity
cHL	classical Hodgkin lymphoma
CLL	chronic lymphocytic leukemia
CML	chronic myeloid leukemia
CRS	cytokine release syndrome
DLBCL	diffuse large B-cell lymphoma
FL	follicular lymphoma
FLT3	fms-like tyrosine kinase 3
GISTs	gastrointestinal stromal tumors
GO	Gemtuzumab ozogamicin
HCC	hepatocellular carcinoma
IMiDs	immunomodulatory drugs
INO	Inotuzumab ozogamicin
ITD	internal tandem duplication
LBCL	large B-cell lymphoma
mAbs	monoclonal antibodies
MAPK	mitogen-activated protein kinase
MCL	mantle cell lymphoma
MM	multiple myeloma
MMAE	monomethylauristatin E
MRD	minimal residual disease
mTOR	mammalian target of rapamycin pathway
MZL	marginal zone lymphoma
NHL	non-Hodgkin lymphoma
PDGFR	platelet-derived growth factor receptor
Ph+ CML	Philadelphia chromosome-positive chronic myeloid leukemia
Ph+ ALL	Philadelphia chromosome-positive acute lymphoblastic leukemia
PI3K	phosphoinositide 3-kinase
PML	progressive multifocal leukoencephalopathy
RCC	renal cell carcinoma
RICs	Radioimmunoconjugates
PROTACs	proteolysis targeting chimeras
SINEs	selective inhibitors of nuclear export
SLL	small lymphocytic lymphoma
TCEs	T-cell engagers
TKIs	tyrosine kinase inhibitors
VEGFR	vascular endothelial growth factor receptor
XPO1	exportin-1
